# Retraction: Tobacco smoking affects the salivary gram-positive bacterial population

**DOI:** 10.3389/fpubh.2025.1654640

**Published:** 2025-07-01

**Authors:** Frontiers Editorial Office

The journal retracts the 19 July 2019 article cited above.

Following publication, it was brought to our attention that the ethical approval for this study referred to the same ethics approval number 2016-011 that had been used across a series of unrelated published studies (1–45). It remains unclear whether appropriate ethics provisions, in line with Frontiers Policies and Publication Ethics policies were adhered to.

The authors and a representative of Aix-Marseille Université Ethics Committee were not able to address all our concerns. As such, this article is retracted.

This retraction was approved by the Chief Executive Editor of Frontiers.

The authors do not agree with this retraction.

## Correction note

This Retraction Notice has been corrected with minor changes to include relevant references.
